# Word order and information structure in Romeyka

**DOI:** 10.3389/fpsyg.2025.1337962

**Published:** 2025-05-19

**Authors:** Nicolaos Neocleous, Ioanna Sitaridou

**Affiliations:** ^1^Aristotle University of Thessaloniki, Greece, United Kingdom; ^2^Faculty of Modern & Medieval Languages and Linguistics Raised Faculty Building University of Cambridge and Queens’ College, Cambridge, United Kingdom

**Keywords:** focus, topic, word order, information structure, OV/VO alternation, headedness, Romeyka, Pontic Greek, Standard Modern Greek

## Abstract

**Introduction:**

This study examines the organization of information structure in Romeyka, the only surviving variety of Asia Minor Greek still spoken in present-day Anatolia, Turkey. Given its historical isolation from Modern Greek and its prolonged contact with Turkish, Romeyka presents a unique linguistic environment for analyzing the structural roles of [focus] and [topic].

**Methods:**

Using empirical data, we investigate how [focus] and [topic] are realized in Romeyka. We analyze their structural positioning within the left periphery and examine their association with an ex situ realization.

**Results:**

Our findings indicate that [focus] and [topic] function as independent structural notions in Romeyka. Both elements are consistently positioned in the left periphery, suggesting a systematic approach to information structuring distinct from Modern Greek.

**Discussion:**

The observed patterns provide evidence of a reorganization of information structure in Romeyka, likely influenced by its long-term linguistic isolation and contact with Turkish. Comparisons with Pontic Greek highlight both similarities and differences, offering insights into the potential contact-induced changes in Romeyka’s grammar.

## 1 Introduction

In this article, we investigate, in turn, two aspects of the grammar of Romeyka^1^, an endangered Asia Minor Greek variety, namely, its word order and information structure. Romeyka is notable for being the only Asia Minor Greek variety still spoken in the Black Sea region of Turkey and as such has attracted significant theoretical interest in recent years, regarding infinitives and complementation, *wh*-formation, double-object constructions, negation, etc. (see Sitaridou, 2013, 2014a, 2014b, 2015, 2017a, 2017b, 2021, 2022, 2023a, 2023b, 2023c; Michelioudakis and Sitaridou, 2013, 2016, 2020, inter alia). However, neither Romeyka’s underlying word order nor the organisation of its information structure has previously been subject to discussion in either descriptive or formal terms.

Specifically, we make two contributions: (i) we provide evidence that Romeyka has underlying head-initial word order in the verbal domain, that is, verb–object (VO), with the verb raising to T^0^; and (ii) we argue for a clausal architecture in Romeyka whereby [topic] and [focus] both constitute autonomous structural notions, realised as projections in the clausal left periphery, and hosting *ex situ* topicalised and focussed constituents, respectively. This study also has a micro-comparative element, as throughout we compare Romeyka with its cognate variety, namely, Pontic Greek (PG) (see Sitaridou and Kaltsa, 2014); we also compare Romeyka with Standard Modern Greek (SMG) and Turkish. This is relevant in view of Romeyka’s socio-historical profile: being spoken in Turkey, Romeyka has for centuries developed in semi-isolation from other Greek varieties, in contact with Turkish instead (see Neocleous, 2022; Neocleous and Sitaridou, 2022; Sitaridou, 2013, 2015).

Previous research has established that Romeyka exhibits both frequent VO and OV order in matrix declarative clauses (see Neocleous, 2020; Neocleous and Sitaridou, 2022; Sitaridou, 2014b; and also Michelioudakis and Sitaridou, 2012, 2013, 2016, 2020; Sitaridou, 2013, 2014a, 2014b, 2015, 2017a, 2017b, 2021, 2022, in prep; Sitaridou and Kaltsa, 2014 for word order in Pontic Greek). Specifically, three permutations of subject (S), verb (V), and object (O) are found in such clauses: SVO (see (1)a), SOV (see (1)b), and OSV (see (1)c), but not V-initial and S-final word orders, namely, VSO (see (2)a), VOS (see (2)b), or OVS (see (2)c) —in contrast to SMG. Subordinate declarative clauses, on the other hand, only permit SOV and OSV when finite (see (3)a-(3)b), though they are obligatorily (S)VO when the embedded verb is non-finite (see (4)).
(1) Matrix declarative clauses:
   a. SVO clause:
      o dohtóris epíren tin aišé.
      the.NOM doctor.NOM marry.PST.3SG the.ACC Ayşe.ACC
      ‘The doctor married Ayşe.’
      (S01; 140102_00080; 01:25)
   b. SOV clause:
      o dohtóris tin aišé epíren.
      the.NOM doctor.NOM the.ACC Ayşe.ACC marry.PST.3SG
      ‘The doctor married Ayşe.’
      (S01; 140102_0008; 01:41)
   c. OSV clause:tin aišé o dohtóris epíren.
      the.ACC Ayşe.ACC the.NOM doctor.NOM marry.PST.3SG
      ‘The doctor married Ayşe.’
      (S01; 140102_0008; 01:33)
(2) Not-attested matrix declarative clauses:
   a. VSO clause:
      ?epíren o dohtóris tin aišé.
      marry.PST.3SG the.NOM doctor.NOM the.ACC Ayşe.ACC
      ‘The doctor married Ayşe.’
   b. VOS clause:
      ?epíren tin aišé o dohtóris.
      marry.PST.3SG the.ACC Ayşe.ACC the.NOM doctor.NOM
      ‘The doctor married Ayşe.’
   c. OVS clause:
      ?tin aišé epíren o dohtóris.
      the.ACC Ayşe.ACC marry.PST.3SG the.NOM doctor.NOM
      ‘The doctor married Ayşe.’
(3) Finite subordinate declarative clauses:
   a. SOV clause:
      o mohalːímis ípen, i ɟylsén aténan utš
      the.NOM teacher.NOM say.PST.3SG the.NOM Gülsen.NOM she.ACC NEG
      aɣapá.
      love.3SG
      ‘The teacher said that Gülsen doesn’t like her.’
      (S02; 812_0065; 05:06)
   b. OSV clause:
      o mohalːímis ípen, aténan i ɟylsén utš
      the.NOM teacher.NOM say.PST.3SG she.ACC the.NOM Gülsen.NOM NEG
      aɣapá.
      love.3SG
      ‘The teacher said that Gülsen doesn’t like her.’
      (S02; 812_0065; 05:01)
(4) Non-finite subordinate declarative clauses:
      (S)VO clause:
      na mutš íχa šíta spundžisíni t ospítin,
      PRT.MOD NEG have.IPFV.1SG clean.INF the.ACC house.ACC
      n épezes me ta χómatæ.
      PRT.MOD play.IPFV.2SG with the.ACC soil.ACC
      ‘If I hadn’t just cleaned the house, you would have played with the soil.’
      (S01; 812_0123; 03:32)

This is like Pontic Greek, in that it also exhibits variation between VO and OV orders. Sitaridou and Kaltsa (2014) argue that this belies an underlying order of VO, with OV orders derived from the discourse-driven fronting of objects. Specifically, they argue that Pontic Greek’s information structure is organised as in (5), with a dedicated landing-site for information focus (IFocP), as well as a higher Contrast projection hosting contrastive topics and foci (ContrastP) and realised by a *pa*-particle, plus up to two dedicated topic positions (TopP).
(5) Pontic Greek:
      TopicP … ContrastP … (TopicP) … IFocP … TP
      
      (Sitaridou and Kaltsa, 2014: p. 23)

In what follows, we will claim that, although Romeyka’s information structure has several properties in common with Pontic Greek, it nonetheless differs from both PG and SMG. On the one hand, we argue Romeyka too has underlying VO order with the verb raising to T^0^, deriving OV from discourse operations; and that, like Pontic Greek but unlike SMG, both focus and topichood are associated with *ex situ* realisations in the left periphery. However, in contrast to Pontic Greek but interestingly parallel to Turkish—the majority and major contact language, we conclude that Romeyka has only a *single* designated focus projection, regardless of the semantic type of focus (information or contrastive); as well as a *single* designated topic position, regardless of the semantic type of topic (aboutness or contrastive).

The article is structured as follows. §2 addresses methodological issues. §3 considers diagnostics for Romeyka’s underlying word order; before a detailed investigation of the distribution of topics and foci, respectively, in Romeyka is presented in §4, offering comparisons with Pontic Greek, SMG, and Turkish as relevant. Drawing on this, §5 then provides a clausal architecture for Romeyka’s left periphery. The article concludes in §6.

## 2 Methodology of data collection

Romeyka is spoken in three enclaves in Turkey’s Black Sea region: Çaykara, Sürmene, and Tonya (see Deffner, 1878; Mackridge, 1987, 1999; Sitaridou, 2013, 2015, 2023a, 2023b, 2023c, 2024; Özkan, 2013; Parcharidis, 1880; Sağlam, 2017; Schreiber and Sitaridou, 2017).

The results reported here were obtained from three corpora consisting of data collected in a remote part of the Çaykara region; specifically, in a village which we refer to as ‘Anasta’ to preserve its anonymity and that of the informants (following Sitaridou, 2013: p. 104). In this study, we use data from two speakers: S01 and S02. Both speakers are female: S01 was in her 40s and S02 in her 70s when interviewed in 2015; the eldest has Romeyka L1 and Turkish L2, and the other one is a more balanced bilingual. In particular, these corpora comprise:

- 34 examples (from S01) from a corpus consisting of data collected by Dr Nicolaos Neocleous during a field trip in the village of ‘Anasta’, Çaykara, Black Sea, Turkey in July 2015, under the guidance, mentoring and supervision of Prof Ioanna Sitaridou who made all arrangements for this field trip and who was there in person for the entire duration, comprising 18 files and amounting to 02:51:43.
- 17 examples (from S01 and S02) from a corpus consisting of audio recordings collected during fieldwork by Prof Ioanna Sitaridou in the village of ‘Anasta’, Çaykara, Black Sea, Turkey in 2012 and 2014, comprising 43 files and amounting to 11:06:11.
- 1 example (from S01) from a corpus consisting of audio recordings collected by Prof Ioanna Sitaridou during a field trip in the village of ‘Anasta’, Çaykara, Black Sea, Turkey in July 2015, comprising 51 files and amounting to 08:25:14.

Throughout this study, we draw on data from all three corpora.^2^ Importantly, the two females are the same in all corpora—first interviewed by Prof. Sitaridou in 2009—so the present corpus consistently describes the grammar of these two speakers. Finally, we also draw data and comparisons on both Romeyka and Pontic Greek—Romeyka’s closest cognate—from a body of previously published studies (see Michelioudakis and Sitaridou, 2012, 2013, 2016; Sitaridou, 2013, 2014a, 2014b, 2015, 2017a, 2017b, 2021, 2022, in prep; Sitaridou and Kaltsa, 2014; Neocleous and Sitaridou, 2022) Crucially, the same grammar has been consistently described by Sitaridou in all her works and collaborations.

The data collection involved oral interviews based on structured questionnaires as well as (semi-)spontaneous data. The data were audio recorded. The audio recordings were transcribed in the International Phonetic Alphabet (IPA) and annotated for the purposes of the study.

## 3 Diagnosing underlying word order: OV or VO?

We focus first on the underlying word order in the verbal domain—that is, whether Romeyka is underlyingly head-initial (VO) or head-final (OV)—before addressing verb placement. Given the availability of both VO and OV orders in matrix clauses attested above (see (1)–(3)), the question of underlying word order is clearly non-trivial. In what follows, we apply several tests to distinguish the relevant possibilities.

A first source of evidence, which forms the basis for several tests, is the word order which surfaces in “pragmatically unmarked” contexts. These are contexts which afford no single constituent a special discourse-oriented interpretation such as topic or focus; such contexts are thus informative because, in principle, they control for discourse-driven movement operations and thus may better reflect the underived, underlying order. One test for pragmatically unmarked order involves ‘all-focus’ questions, exemplified by ‘What happened?’. Since these typically involve a context in which all the information expressed in the answer constitutes new information, no *single* constituent has a special discourse-oriented interpretation—all are equally focussed. Thus, the answer is ‘pragmatically unmarked’ (Büring, 2009; van der Wal, 2016). Importantly, this test yields VO order as pragmatically unmarked in Romeyka (see (6)), just as it does for SMG, a language trivially analysed as underlyingly VO (see (7)).(6) Romeyka:
   a. Question: do eʝéndo?
      what.NOM happen.PST.3SG
      ‘What happened?’
   b. Answer:
      o mustafás epelæpsen to χoráfin.
      the.NOM Mustafas.NOM put.fertiliser.PST.3SG the.ACC field.ACC
      ‘Mustafas put fertiliser on the field.’
      (S01; 150703_0040; 02:16)
(7) Modern Greek:
   a. Question:
      ‘What happened?’
   b. Answer:
      éspase ti lába o ʝánis.
      break.PST.3SG the.ACC lamp.ACC the.NOM Yanis.NOM
      ‘Yanis broke the lamp.’
      
      Alexiadou and Anagnostopoulou

Further evidence that Romeyka’s underived word order is VO comes from the behaviour of non-finite subordinate clauses. As above, such clauses are obligatorily VO (see (4)); that is, the matrix clause auxiliary verb must precede the non-finite embedded verb, which must precede the object of the embedded clause, yielding Aux-V-O, as in (8). Given that *only* VO is licit in this context it is plausible to assume that VO is the syntactically unmarked, i.e., basic word order; that is, Romeyka is underlyingly VO. Crucially, in Cappadocian, we find the opposite pattern, namely Aux-O-V orders (see Neocleous and Sitaridou, 2022).(8) Romeyka:
      n íχame paníni χtisíni t ospít
      PRT.MOD have.PST.3PL go.INF build.INF the.ACC house.ACC
      so parχár!
      in.the.ACC pasture.ACC
      ‘I wish we had gone to build the house in the highland pastures!’
      
      (Sitaridou, 2014b: p. 136)

A second question more indirectly related to word order, is the position in which the verb surfaces. We present evidence that it surfaces in T^0^, at least in matrix declarative clauses; that is, Romeyka exhibits V^0^-to-T^0^ raising. In this way, Romeyka patterns with SMG, which is standardly held to exhibit verb-raising.

To start with, Romeyka exhibits two typological traits which at least frequently correlate with V^0^-to-T^0^ raising; this is evidence, albeit only suggestive, in favour of a raising analysis. On the one hand, Romeyka shows rich person and number agreement; this is consistent with the Rich Agreement Hypothesis (see Pollock, 1989; Roberts, 1993; Vikner, 1995, 1997), the generalisation that V^0^-to-T^0^ raising correlates with and according to the RAH is conditioned by, rich subject agreement on the finite verb. For example, Romeyka has distinct verbal forms for all persons, and singular and plural numbers with no suppletion, at least in most tense–voice combinations. Table 1 shows the present active declension of *léɣo* ‘I say’.

**Table 1:** Subject agreement paradigm in Romeyka.

Person	Singular number	Plural number
1st	léɣ-o	léɣ-umen
2nd	léʝ-is	léʝ-ete
3rd	léʝ-i	léɣ-un(e)

However, the RAH has been disputed: empirically, on the grounds that even poorly inflected languages have been claimed to exhibit V^0^-to-T^0^ movement; and theoretically, because contemporary post-syntactic insertion models of morphology dilute its ability to influence syntax, and thus rule out rich morphology directly conditioning syntactic movement (though see Koeneman and Zeijlstra, 2014 for a rebuttal). Nonetheless, since a(n imperfect) correlation holds, this evidence is still suggestive. It is also informative that Romeyka has high tense synthesis, since on Biberauer and Roberts’ (2010) alternative account, it is tense synthesis which correlates with verb-raising instead; again, suggesting evidence for verb-raising in Romeyka.

The same is true of the second typological trait, pro-drop, whereby a clause contains no overt subject (see (9)).(9) Romeyka:
      opsé χars ípe tes.
      yesterday now say.PST.3SG she.ACC
      ‘Yesterday she immediately told her.’
      (S01; 0120713192027; 01:36)


Several accounts of pro-drop postulate an indirect relationship with V^0^-to-T^0^. Alexiadou and Anagnostopoulou (1998), for example, propose that verb movement to T^0^ is sufficient to identify T^0^’s formal features, voiding the requirement that a subject occupy [Spec,TP] (the Extended Projection Principle of Chomsky, 1982), so permitting pro-drop. Approaches of this sort are problematic in view of recent theoretical developments: as Holmberg (2005) points out, if verb-raising is enough to value T^0^’s phi-features, then these must be base-generated as valued either on V^0^ or T^0^—unexpected if semantically uninterpretable features are always base-generated as *un*valued as in Chomsky (2001). Nonetheless, to the extent that there is again an empirical correlation between verb-raising and pro-drop, this is again suggestive evidence that Romeyka has verb-raising.

A more robust argument for V^0^-to-T^0^ raising comes from placement facts. Consider the additive particle *dže* ‘also’. *dž(e)* is like Modern Greek *ce* ‘also’: it is a focal associate operator which surfaces only in a base-generated position as a sister to its associate; we take this position to be [Spec, vP], following Chatzikyriakidis et al.’ (2015) analysis of *ce*. Crucially, as example (10) shows, finite verbs precede *dž(e)*—providing strong evidence that the verb must raise out of vP, i.e., to T^0^, and thus past *dž(e)*.(10) Romeyka:
      Context:
      *eftǽmen vútiron. ta tsupaðítikæ t alévræ ɣavurévumen. θénumen dže neron. θénumen dže álas. evrázumen to nerón.* “We add butter, we fry the flour in the butter, we also put water, we also put salt, we boil the water.”
      θénumen dže álas.
      put.1PL PRT salt.ACC
      ‘We also put salt.’
      (S01; 150703_0041; 05:09)


Thus, we conclude that Romeyka does exhibit V^0^-to-T^0^ movement; and, on the strength of the evidence above, that it has underlying VO word order.

## 4 Information structure: distribution of topics and foci in Romeyka

We currently turn to the second goal of this article: to probe the organisation of information structure in Romeyka. This will explain, among other things, the existence of those other pragmatically marked, OV word orders. In this section, we consider, in turn, the distributions of topics and then foci in Romeyka, comparing this in each case with Romeyka’s cognate variety, Pontic Greek. This evidence forms the basis for our proposal for the architecture of the left periphery in Romeyka, discussed in §5.

### 4.1 Topics

We begin with topics, concentrating specifically on topics of two kinds: aboutness topics and contrastive topics. Aboutness topics are identified in the literature as the constituent which represents the theme of the predication, i.e., what the sentence is about (see Frascarelli and Hinterhölzl, 2007). A contrastive topic, on the other hand, is the sort of interpretation favoured for a constituent in a context where the hearer answers a question differing from the one being asked; that is, the constituent contrasts with some contextually salient alternative (see Büring, 2003, 2009).

Romeyka employs four syntactic strategies to convey topics in discourse. First, a constituent may be realised *ex situ*. Specifically, it may be left-dislocated, appearing preverbally and interpreted either as an aboutness (11) or a contrastive topic (12). This is unlike SMG, for example, which largely restricts left dislocation to contrastive constituents (see §5 below but see Gryllia, 2008 for a different view).(11) Romeyka:
   a. Question: tin aišén ts epíren?
      the.ACC Ayşe.ACC who.NOM marry.PST.3SG
      ‘Who married Ayşe?’
   b. Answer:
      [tin aišén]_A-Top_ o mohalímis epíren.
      the.ACC Ayşe.ACC the.NOM teacher.NOM marry.PST.3SG
      ‘The teacher married Ayşe.’
      (S01; 140102_0008; 01:10)
(12) Romeyka:
   a. Context: to pontʰólin alís epíren,
      the.ACC trousers.ACC Alis.NOM buy.PST.3SG
      to kazáçin o mehmétis epíren.
      the.ACC sweater.ACC the.NOM Mehmetis.NOM buy.PST.3SG
      ‘Alis bought the trousers and Mehmetis bought the sweater.’
   b. Question: to pont^h^ólin ts epíren
      the.ACC trousers.ACC who.NOM buy.PST.3SG
      tše to kazáçin ts epíren?
      and the.ACC sweater.ACC who.NOM buy.PST.3SG
      ‘Who bought the trousers and who the sweater?’
   c. Answer:
      [to pontʰólin]_C-Top_ alís epíren,
      the.ACC trousers.ACC Alis.NOM buy.PST.3SG
      áma [to kazáçin]_C-Top_ o mehmétis epíren.
      but the.ACC sweater.ACC the.NOM Mehmetis.NOM buy.PST.3SG
      ‘Alis bought the trousers, but Mehmetis bought the sweater.’
      (S01; 150702_0014; 05:10)

Second, an aboutness topic —but not a contrastive topic (see (13)b)— may be yielded through clitic left dislocation (ClLD) with the only clitic attested in Romeyka (see Sitaridou, 2017b), i.e., *æ* ‘him/her/it/them’ (see (13)a):(13) Romeyka:
   a. ombrón [ta patsíðæ]_A-Top_, in.the.past the.ACC girls.ACC
       s okʰúlːin tš epóliɣan æ.
      to school.ACC NEG send.IPFV.3PL them
      ‘In the past, they did not send the girls to school.’
      (S01; 150702_0019; 03:23)
   b. ?ombrón [ta  patsíðæ]_C-Top_, in.the.past  the.ACC girls.ACC,
       s okʰúlːin tš epóliɣan æ.
      to school.ACC NEG send.IPFV.3PL them
      ‘In the past, they did not send the girls to school.’

Interestingly, although ClLD also occurs in SMG, it does not have the same pragmatic import as in Romeyka. While in SMG a left-dislocated constituent is interpreted as a topic if and only if it is ClLD’ed (see (14)a), otherwise being interpreted as a focus (see (14)b), in Romeyka a left-dislocated constituent (even if it is a definite DP) can be interpreted as a topic even if it is not ClLD’ed (see a ClLD’ed topic in (15)a and a non-ClLD’ed one in (15)b. ClLD’ed topics in Romeyka cannot be C-Top (see (15)c):(14) Modern Greek:
   a. [to ʝáni]_Top_, ton sinádisa χθes. the.ACC Yanis.ACC he.ACC meet.PST.1SG yesterday
      ‘I met Yanis yesterday.’
   b. [to ʝáni]_Foc_, (*ton) sinádisa  χθes. the.ACC Yanis.ACC he.ACC meet.PST.1SG yesterday
      ‘It is Yanis that I met yesterday.’

      (Tsimpli, 1995: p. 179)


(15) Romeyka:
   a. [ta patátes]_A-Top_ zimónum æ. the.ACC potatoes.ACC knead.1PL them
      ‘We knead the potatoes.’
      (S01: 150702_0019; 05:52)
   b. Context:
      *tróɣum ata. eftǽm æ me ta patátes. me ta ʝeralmasíæ. kaθarízum æ. ta ʝeralmasíæ kuzardévum æ.* “We eat them. We make them with potatoes. With potatoes. We peel and slice the potatoes.”
      [ta patátes]_A-Top_ zimónum.
      the.ACC potatoes.ACC knead.1PL
      ‘We knead the potatoes.’
      (S01: 150702_0019; 06:25)
   c. [ta patátes]_C-Top_ zimónum æ.
      the.ACC potatoes
       ACC knead.1PL them
      ‘We knead the potatoes.’


The third strategy is the use of a topic particle, i.e., *pa(l)*. This assigns contrastive (but not aboutness—see (16)b) topichood to the constituent with which it is associated (see (16)a):(16) Romeyka:
   a. eɣó [ton p^h^ará pal]_C-Top_ ðíɣo se.
      I the.ACC money.ACC PRT give.1SG you.ACC
      ‘I give you the money.’
      (S01; 0120713192027; 01:49)
   b. ?eɣó [ton p^h^ará  pal]_A-Top_ ðíɣo se.
      I the.ACC money.ACC PRT  give.1SG you.ACC
      ‘I give you the money.’

Fourth, given information *may* appear postverbally, but only if non-contrastive (see (17)):(17) Romeyka:
   a. Question:
      Píos epíren tin aišén?
      who.NOM marry.PST.3SG the.ACC Ayşe.ACC
      ‘Who married Ayşe?’
   b. Answer:
      o dohtóris epíren tin aišén.
      the.NOM doctor.NOM marry.PST.3SG the.ACC Ayşe.ACC
      ‘The doctor married Ayşe.’
      (S01; 140102_0008; 01:25)

In sum, then, contrastive topics in Romeyka must be realised *ex situ* in preverbal position though they may also be marked with the *pa(l)* topic particle; non-contrastive given information, like aboutness topics, may also be realised *ex situ*, though can also be encoded *in situ* or with ClLD.

This is similar in important respects to Pontic Greek. Pontic Greek has *two* main strategies for conveying old information, both of which have parallels in Romeyka: (a) ClLD and (b) usage of a particle, *pa* (Sitaridou and Kaltsa, 2014: p. 6). ClLD is exemplified by (18)a-b. Interestingly, Pontic Greek and Romeyka also both show the same prohibition on clitic doubling with right dislocation, not found in SMG (Sitaridou and Kaltsa, 2014: p. 6); compare Pontic Greek (18)c-d with Romeyka (19)b.(18) Pontic Greek:
   a. tin elean eðek aten to ʝitonan. the.ACC olive.ACC give.PST.1SG her the.ACC neighbour.ACC
      ‘I gave the olive to the neighbour.’
   b. ?ton ʝitonan   eðek aton din elean. the.ACC neighbour.ACC give.PST.1SG he.ACC the.ACC olive.ACC
   c. *eðek aten to ʝitonan din elean. give.PST.1SG her the.ACC neighbour.ACC the.ACC olive.ACC
      d. *eðek aton to ʝitonan din elean. give.PST.1SG he.ACCthe.ACC neighbour.ACC the.ACC olive.ACC

      (Sitaridou and Kaltsa, 2014: p. 6 *apud* Drettas, 1997: p. 278)

(19) Romeyka:
   a. ta patátes zimónum æ. the.ACC potatoes.ACC knead.1PL them
      ‘We knead the potatoes.’
      (S01: 150702_0019; 05:52)
   b. zimónum æ ta patátes. knead.1PL them the.ACC potatoes.ACC
       ‘We knead the potatoes.’

The *pa*-particle, on the other hand, attaches at the right edge of a (contrastively) topicalised constituent. The *pa*-marked constituent must also be realised *ex situ* in preverbal position as *tin aðelfi s pa* ‘your sister’ is in (20). This is again like Romeyka, which we have shown permits *ex situ* topics, as (21) again attests.(20) Pontic Greek: tin aðelfi s  pa m æɣliɣoris.
      the.ACC sister.ACC you.POSS PRT NEG forget.2SG
      ‘As for your sister, don’t rush (into marrying her).’

      (Sitaridou and Kaltsa, 2014: p. 8 apud Melanofrydis, 2001: p. 13)(21) Romeyka:
      eɣó ton p^h^ará  pal ðíɣo se.
      I the.ACC money.ACC PRT give.1SG you.ACC
      ‘I give you the money.’
      (S01; 0120713192027; 01:49)

However, Pontic Greek’s *pa* and Romeyka’s *pa(l)* are not exactly analogous. As discussed at length by Sitaridou and Kaltsa (2014), *pa* functions as a contrastive particle in Pontic Greek, realising the head of a ContrastP projection in the left periphery. Romeyka’s *pa(l)*, conversely, does not seem to encode contrast, being more rigidly associated with topichood (instead of contrast). Rather, *pa(l)* seems to reflect the stage prior to the one we find in Pontic Greek, where grammaticalisation of contrast into the particle has not occurred; Pharasiot and Rumeic Greek also seem to instantiate this stage (see Agouraki, 2010; Dawkins, 1916; Kisilier, 2007). Given this, we might consider Romeyka’s *pa(l)*-phrases to instantiate the head of a dedicated TopP instead, rather than ContrastP.

### 4.2 Information foci

We currently turn to the distribution of focussed constituents, starting with information focus. Focus can be defined as the part of the sentence which is not presupposed (Jackendoff, 1972; Chomsky, 1972). The information focus constitutes the assertion of an utterance, i.e., its non-presupposed content, without any further restrictions; it simply asserts the membership of an individual in a set (see Gundel, 1998).

The most widely accepted test for focussed constituents is to use *wh*-questions and their answers (Beaver and Clark, 2008; Krifka, 2007; Lambrecht, 1994; Rooth, 1992; van der Wal, 2016, i.a.). A *wh*-question always yields new information, relating to the *wh*-questioned constituent; accordingly, if focus is defined as the new (i.e., non-presupposed) information in a sentence, then it follows that the phrase that replaces the *wh*-constituent is focussed.

We apply this test to Romeyka in (22) and (23). These examples clearly demonstrate that Romeyka forces information-focussed constituents to appear preverbally; the focussed objects *χavíts* ‘pudding’ and *pol:á chitápæ* ‘many books’ both appear *ex situ*, immediately left adjacent to the verb. Indeed, the infelicity of (22)c and (23)c, with the focussed constituents in postverbal position, suggests that the preverbal realisation is obligatory.(22) Romeyka:
   a. Question:
      alís dóɣna éfaen?
      Alis.NOM what.ACC eat.PST.3SG?
      ‘What did Alis eat?’
      Answers:
   b. alís [χavíts]_I-Foc_ éfaen.
      Alis.NOM pudding.ACC eat.PST.3SG
      ‘Alis ate a pudding.’
      (S01; 150703_0040; 07:14)
   c. #alís éfaen [χavíts]_I-Foc_. Alis.NOM eat.PST.3SG pudding.ACC
      ‘Alis ate a pudding.’
(23) Romeyka:
   a. Question:
      dó eχúʝepsen?
      what.ACC read.PST.3SG
      ‘What did s/he read?’
      Answers:
   b. [polːá c^h^iTÁpæ]_I-Foc_ eχúʝepsen. many.ACC books.ACC read.PST.3SG
      ‘S/He read many books.’
      (S01; 812_0059; 00:10)
   c. #eχúʝepsen [polːá c^h^iTÁpæ]_I-Foc_.read.PST.3SG many.ACC books.ACC
      ‘S/He read many books.’

This is unlike SMG, in which information focus has traditionally been said to occur only postverbally, as (24) and (25) suggest.(24) Modern Greek:
   a. Question: ti éfaʝe o ʝórɣos?
      what.ACC eat.PST.3SG the.NOM George.NOM
      ‘What did George eat?’
   b. Answer:
      o ʝórɣos éfaʝe [tin kobósta]_I-Foc_. the.NOM Geroge.NOM eat.PST.3SG the.ACC stewed-fruit.ACC
      ‘George ate the stewed fruits.’

      (Sitaridou and Kaltsa, 2014: p. 12)(25) Modern Greek:
   a. Question: ti ðʝávase?
      what.ACC read.PST.3SG
      ‘What did s/he read?’
      Answers:
   b. ðʝávase [polá vivlía]_I-Foc_. read.PST.3SG many.ACC books.ACC
      ‘S/He read many books.’
   c. ?[polá vivlía]_I-Foc_ ðʝávase. many.ACC books.ACC read.PST.3SG
      ‘S/He read many books.’

      (Sitaridou and Kaltsa, 2014: p. 12)

This difference attenuates if we consider Gryllia’s (2008) findings, which show—based on experimental tests—that preverbal objects are neither necessarily exhaustive nor exclusively contrastive in SMG; such that information focus can be preverbal. For example, the focussed direct object is interpreted as a new information focus both when occurring in OV order, and in VO order in (26)c and (26)b, respectively.(26) Modern Greek:
   a. Question: ti χárise metaksí álon o ʝánis
      what.ACC give.PST.3SG among others.GEN o ʝánis stin ilektra?  the.NOM Yanis.NOM      to.the.ACC Ilektra.ACC      ‘Among other things, what did Yanis give to Ilektra?’      Answers:
   b. χárise [éna vivlío]_I-Foc_ stin iléktra.
      give.PST.3SG a.ACC book.ACC to.the.ACC Ilektra.ACC
      ‘He gave a book to Ilektra.’
   c. [éna vivlío]_I-Foc_ χárise stin iléktra.
   a.ACC book.ACC give.PST.3SG to.the.ACC Ilektra.ACC
      ‘He gave a book to Ilektra.’

      (Gryllia, 2008: p. 21)

Nevertheless, the fact that SMG may in fact allow *both* options does not alter the parametric difference with Romeyka, where the preverbal position is the only option. This is particularly clear in the following judgement made by a Romeyka speaker in (27).(27) Romeyka:
   a. Question:
      ánda erotó se alís dóɣna éfaen
      if ask.1SG you.ACC Alis.NOM what.ACC eat.PST.3SG
      esí léʝis me, o alís éfaen míla
      you.NOM say.2SG I.ACC the.NOM Alis.NOM eat.PST.3SG apples.ACC
      ʝóksa, alís míla éfaen?
      or Alis.NOM apples.ACC eat.PST.3SG
      ‘If I ask you, what did Alis eat, what do you say to me? Alis ate apples, or Alis apples ate?’
   b. Answer:
      kalːíon, alís [míla]_I-Foc_ éfaen.
      better Alis.NOM apples.ACC eat.PST.3SG
      ‘Alis ate apples, sounds better.’
      (S01; 812_0055; 03:09)

By contrast, Pontic Greek does pattern with Romeyka: information-focussed constituents in Pontic Greek also occur preverbally as demonstrated by *to χošaf* ‘the stewed fruit’ in (28) and *pola vivlia* ‘many books’ in (29) below.(28) Pontic Greek:
   a. Question:
      o ʝorikas do efaen?
      the.NOM Yorikas.NOM what.ACC eat.PST.3SG?
      ‘What did Yorikas eat?’
      Answers:
   b. (o ʝorikas) [to χošaf]_I-Foc_ efaen. the.NOM Yorikas.NOM the.ACC stewed.fruit.ACC eat.PST.3SG
      ‘Yorikas ate stewed fruit.’
   c. #o ʝorikas efaen [do χošaf]_I-Foc_. the.NOM Yorikas.NOM eat.PST.3SG the.ACC stewed.fruit.ACC
      ‘Yorikas ate stewed fruit.’

      (Sitaridou and Kaltsa, 2014: p. 12)(29) Pontic Greek:
   a. Question: do eðevasen?
      what read.PST.3SG?
      ‘What did he read?’
      Answers:
   b. pola vivlia eðevasen. many.ACC books.ACC read.PST.3SG
      ‘He read many books.’
   c. #eðevasen pola vivlia. read.PST.3SG many.ACC books.ACC
      ‘He read many books.’

      (Sitaridou and Kaltsa, 2014: p. 12)

This suggests information focus in Romeyka may be consistent with the conclusion reached in recent research on its Pontic Greek counterpart, namely, that it appears in the left periphery (see Sitaridou and Kaltsa, 2014). For example, in both Romeyka and Pontic Greek any focussed phrase—no matter the phrase type—appears before the verb: direct object (NP) (see (30) from Romeyka and (31) from Pontic Greek), direct object (DP) (see (32) from Romeyka and (33) from Pontic Greek), indirect object (beneficiary) (DP) (see (34) from Romeyka and (35) from Pontic Greek), predicative (adjective) (see (36) from Romeyka and (37) from Pontic Greek), adverbial (NP) (see (38) from Romeyka and (39) from Pontic Greek), and existential constructions (see (40) from Romeyka and (41) from Pontic Greek):(30) Romeyka:
      Direct object (NP) is focussed:
   a. Question:
      alís dóɣna éfaen?
      Alis.NOM what.ACC eat.PST.3SG
      ‘What did Alis eat?’
   b. Answer:
      alís [χavítsin]_I-Foc_ éfaen.
      Alis.NOM pudding.ACC eat.PST.3SG
      ‘Alis ate a pudding.’
      (S01; 150703_0040; 07:14)
      (31) Pontic Greek:
      Direct object (NP) is focussed:
   a. Question: do efaes?
      what.ACC eat.PST.2SG
      ‘What did you eat?’
   b. Answer:
      [χavits]_I-Foc_ efaa.
      pudding.ACC eat.PST.1SG
      ‘I ate pudding.’

      (Sitaridou and Kaltsa, 2014: p. 13 apud Drettas, 1997: p. 280)(32) Romeyka:
      Direct object (DP) is focussed:
   a. Question:
      i aišé tínan epíren?
      the.NOM Ayşe.NOM who.ACC marry.PST.3SG
      ‘Who did Ayşe marry?’
   b. Answer:
      i aišé [ton dohtórin]_I-Foc_ epíren.
      the.NOM Ayşe.NOM the.ACC doctor.ACC marry.PST.3SG
      ‘Ayşe married the doctor.’
      (S01; 140102_0008; 01:37)
(33) Pontic Greek:
      Direct object (DP) is focussed:
   a. Question: do eplises?
      what.ACC wash.PST.2SG
      ‘What did you wash?’
   b. Answer:
      [ta poðaræ m]_I-Foc_ eplisa.
      the.ACC feet.ACC I.POSS wash.PST.1SG
      ‘I washed my feet.’

      (Sitaridou and Kaltsa, 2014: p. 13 apud Drettas, 1997: p. 280)(34) Romeyka:
      Indirect object (beneficiary) (DP) is focussed:
   a. Question: to c^h^itápin tínan éndžes?
      the.ACC book.ACC who.ACC bring.PST.2SG
      ‘To whom did you give the book?’
   b. Answer: to c^h^itápin [ton ʝuSÚfin]_I-Foc_ éŋga.
      the.ACC book.ACC the.ACC Yusufis.ACC bring.PST.1SG
      ‘I brought the book for Yusufis.’
      (S01; 150703_0042; 00:54)
(35) Pontic Greek:
      Indirect object (beneficiary) (DP) is focussed:
      epita ti nifæn θa eniγane lutron.
      then the.ACC bride.ACC PRT.FUT open.IPFV.3PL bath.ACC
      ‘Then they would prepare the bath for the married girl.’

      (Sitaridou and Kaltsa, 2014: p. 13 apud Drettas, 1997: p. 280)(36) Romeyka:
       Predicative (adjective) is focussed:
   a. Question:
      alís do en?
      Alis.NOM what.NOM be.3SG
      ‘What is Alis?’
   b. Answer:
      alís [áɣuros]_I-Foc_ en.
      Alis.NOM boy.NOM be.3SG
      ‘Alis is a boy.’
      (S01; 140102_0009; 00:20)
(37) Pontic Greek:
      Predicative (adjective) is focussed:
   a. Question: do en atos?
      what.ACC be.3SG  he.NOM
      ‘What is he like?’
   b. Answer: palalos en.
      crazy.NOM be.3SG
      ‘He is crazy.’

      (Sitaridou and Kaltsa, 2014: p. 14 apud Drettas, 1997: p. 555)(38) Romeyka:
      Adverbial (NP) is focussed:
   a. Question:
      i mána s póte efáise ton musafírin?
      the.NOM mother.NOM you.POSS when feed.PST.3SG the.ACC guest.ACC
      ‘When did your mother feed the guest?’
   b. Answer:
      [opsé]_I-Foc_ efáisen ton musafírin.
      yesterday feed.PST.3SG the.ACC guest.ACC
      ‘She fed the guest yesterday.’
      (S01; 150703_0041; 07:10)
(39) Pontic Greek:
      Adverbial (NP) is focussed:
      mesaniχts eton.
      midnight be.PST.3SG
      ‘It was midnight.’

      (Sitaridou and Kaltsa, 2014: p. 14 *apud* Drettas, 1997: p. 555)(40) Romeyka:
      Existential construction is focussed:
   a. Question:
      o šcʰílːon do en?
      the.NOM dog.NOM what.NOM be.3SG
      ‘What is the dog?’
   b. Answer:
      [haivánin]_I-Foc_ en.
      animal.NOM be.3SG
      ‘It’s an animal.’
      (S01; 140102_0009; 00:35)
(41) Pontic Greek:
      Existential construction is focussed:
      χorafæ  c^h^ ine.
      fields NEG exist.3PL
      ‘There are no fields.’

      (Sitaridou and Kaltsa, 2014: p. 14)

Second, the two varieties pattern together in having focus-fronting in questions of “total ignorance” that yield a yes/no reply (Sitaridou and Kaltsa, 2014: p. 14). See (42) from Romeyka and (43) from Pontic Greek:(42) Romeyka:
      esís [ta tsupáðæ]_I-Foc_ θerízete?
       you.NOM the.ACC corn.ACC harvest.2PL
       ‘Do you harvest the corn?’
      (S02; 812_0067; 01:58)
(43) Pontic Greek:
   a. Question:
      t apiðæ ekserts?
      the.ACC pears.ACC know.2SG
      ‘Do you know the pears?’
   b. ???ekserts t apiðæ? know.2SG the.ACC pears.ACC
      ‘Do you know the pears?’

      (Sitaridou and Kaltsa, 2014: p. 14)

Third, just like Romeyka, Pontic Greek requires strict adjacency between the fronted information-focussed constituent and the predicate it precedes, especially where the predicate is the verb *be* or *have* (Sitaridou and Kaltsa, 2014: p. 14). In (44), for example, the adverb *panda* ‘always’ cannot interpolate between the information focussed *aiksa* ‘like this’ and the verb *esne* ‘were’, otherwise infelicity ensues (44)b.(44) Pontic Greek:
   a. aiksa esne panda. like.this be.IPFV.2SG always
      ‘You were always like this.’
   b. *aiksa panda esne. like.this always be.IPFV.2SG
      ‘You were always like this.’

      (Sitaridou and Kaltsa, 2014: p. 14 apud Drettas, 1997: p. 182)

Fourth, both varieties permit movement of the focussed constituent in subordinate clauses as in (45) in Romeyka and (46) in Pontic Greek (Sitaridou and Kaltsa, 2014: p. 14). Thus, the behaviour of information focus in Romeyka and Pontic Greek is highly consistent.(45) Romeyka:
   a. Question: do θarís, alís tínan efílisen?
      what.ACC think.2SG Alis.NOM who.ACC kiss.PST.3SG
      ‘Who do you think that Alis kissed?’
   b. Answer:
      eɣó θaró, alís [tin aišén]_I-Foc_ efílisen.
      I think.1SG Alis.NOM the.ACC Ayşe.ACC kiss.PST.3SG
      ‘I think that Alis kissed Ayşe.’
      (S01; 150703_0040; 19:07)
(46) Pontic Greek:
   a. eθaresen oti tšantarmas eton. think.PST.3SG that policeman be.IPFV.3SG
      ‘He thought (that) he was a policeman.’
   b. eθaresen džantarmas eton. think.PST.3SG policeman be.IPFV.3SG
      ‘He thought he was a policeman.’

      (Sitaridou and Kaltsa, 2014: p. 15 apud Drettas, 1997: p. 370)

### 4.3 Contrastive foci

Consider finally *contrastive* focus. This involves the selection of a subset from a set of alternatives, *contrasting* with a contextually salient individual (see Molnár, 2006).

In Romeyka, contrastive focus patterns like information focus: the focussed constituent occurs preverbally as in (47). Indeed, as the Romeyka speaker’s grammaticality judgement in (48) suggests, this is the *only* option for encoding contrastive focus; again, this is like information focus.(47) Romeyka:
   a. Question:
      kahVÉN ʝóksa tšáin θélis?
      coffee.ACC or tea.ACC want.2SG
      ‘Do you want coffee or tea?’
      Answers:
   b. eɣó [kahvén]_C-Foc_ θélo.
      I.NOM coffee.ACC want.1SG
      ‘I want coffee.’
      (S01; 150702_0013; 12:15)
   c. manaχón [kahvén]_C-Foc_ thelo. only coffee.ACC want.1SG
      ‘I only want coffee.’
      (S01; 150702_0013; 12:22)
(48) Romeyka:
   a. Question:
      eɣó léɣo se alís apʰíðæ aɣórasen,
      I.NOM say.1SG you.ACC Alis.NOM pears.ACC buy.PST.3SG
      áma esí eksérts alís míla aɣórasen.
      but you.NOM know.2SG Alis.NOM apples.ACC buy.PST.3SG
      eɣó érχome léɣo se alís apʰíðæ aɣórasen.
      I.NOM come.1SG say.1SG you.ACC Alis.NOM pears.ACC buy.PST.3SG
      esí dóɣna léʝis me?
      you.NOM what.ACC say.2SG I.ACC?
      ‘Alis bought pears, but you know that he bought apples. I came and told you that Alis bought pears. What do you reply to me?’
   b. Answer:
       alís [míla]_C-Foc_ aɣórasen.
      Alis.NOM apples.ACC buy.PST.3SG
       ‘Alis bought apples.’
      (S01; 812_0055; 01:54)

Any type of phrase can be contrastively focussed in Romeyka, just as it can be information focussed: object (NP) (see (49)), object (DP) (see (50)), predicative complement (see (51)), adverbial phrase (see (52)), among others (see Neocleous, 2020: p. 160ff):(49) Romeyka:
       Direct object (NP) is focussed:
   a. Question:
      o mehmétis míla ʝóksa aP^H^Íðæ aɣórasen?
      the.NOM Mehmetis.NOM apples.ACC or pears.ACC buy.PST.3SG
      ‘Did Mehmetis buy apples or pears?’
   b. Answer:
      o mehmétis [míla]_C-Foc_ aɣórasen.
      the.NOM Mehmetis.NOM apples.ACC buy.PST.3SG
      ‘It’s apples that Mehmetis bought.’
      (S01; 150702_0013; 12:05)
(50) Romeyka:
      Direct object (DP) is focussed:
   a. Question:
      o ramazánis ti zeiNÉP epíren?
      the.NOM Ramazanis.NOM the.ACC Zeynep.ACC marry.PST.3SG
      ‘Did Ramazanis marry Zeynep?’
   b. Answer:
      o ramazánis [tin aišén]_C-Foc_ epíren.
      the.NOM Ramazanis.NOM the.ACC Ayşe.ACC marry.PST.3SG
      ‘It’s Ayşe that Ramazanis married.’
       (S01; 140102_0009; 07:50)
(51) Romeyka:
      Predicative complement is focussed:
   a. Question:
      dóɣna en avúto? vútiron?
      what.NOM be.3SG this.NOM butter.NOM
      ‘What is this? Butter?’
   b. Answer(s):
      [anθóɣalan]_C-Foc_ en.
      buttermilk.ACC be.3SG
      ‘This is buttermilk.’
      (S01; 812_0055; 00:54)
(52) Romeyka:
      Adverbial phrase is focussed:
   a. Question:
      alís osímːeron érθen asin tšáikaran?
      Alis.NOM today come.PST.3SG from.the.ACC Çaykara.ACC
      ‘Did Alis come from Çaykara today?’
   b. Answer:
       ʝokʰ, [opsé]_C-Foc_ érθen.
      no yesterday come.PST.3SG
      ‘No, he came yesterday.’
      (S01; 150703_0040; 08:46)

Unlike in the case of information focus, SMG typically realises contrastive focus by left dislocation to a preverbal position —so patterns with Romeyka. This is exemplified by (53). This is also true of Pontic Greek, as in (54).(53) Modern Greek:
   a. Question:
      θélis kaFÉ i TSÁI?
      want.2SG coffee.ACC or tea.ACC
      ‘Do you want coffee or tea?’
       Answers:
   b. [kaFÉ]_C-Foc_ θélo.coffee.ACC want.1SG
      ‘I want coffee.’
   c. móno [kaFÉ]_C-Foc_ θélo. only coffee.ACC want.1SG
      ‘I only want coffee.’

      (Sitaridou and Kaltsa, 2014: p. 12)

(54) Pontic Greek:
   a. Question:
      θelts na pseno se gaiven
      want.2SG PRT.MOD make.1SG you.ACC coffee.ACC
      ci ena ðio otia na vukuse?
      and one.ACC two.ACC sweets.ACC PRT.MOD dunk.PNP.2SG
      ‘Do you want me to make you some coffee and a couple of sweets to dunk in the coffee?’
      Answers:
   b. kaiven pseson.
      coffee.ACC make.IMP.2SG
      ‘Make coffee (and not something else).’
      b’. manaχon kaiven pseson.
      only coffee.ACC make.IMP.2SG
      ‘Only make coffee.’
   c. *manaχon kaiven pa pseson.
      only coffee.ACC PRT make.IMP.2SG
      ‘Only make coffee.’
      d. kaiven pa θelo.
      coffee.ACC PRT want.1SG
      ‘I want coffee.’
      e. kaiven pa θelo, otia pa θelo.
      coffee.ACC PRT want.1SG sweets.ACC PRT want.1SG
      ‘I want both coffee and cookies.’

      (Sitaridou and Kaltsa, 2014: pp. 11–12)

Contrastive focus in Pontic Greek nonetheless differs from Romeyka in at least two regards. First, though it can encode contrastive focus by focus movement to preverbal position, the contrastive focussed constituent need not be strictly adjacent to the predicate. Thus, in (55) below, the left periphery elements (topicalised *aika emorfa peðja* ‘such beautiful children’, focalised *esis* ‘you’) are separated from the verb by the adverb *kamian* ‘never’, violating strict adjacency (Sitaridou and Kaltsa, 2014: p. 15). This contrasts with Romeyka, in which contrastive focussed constituents are obligatorily strictly adjacent to the predicate. This is parallel to the behaviour of information-focussed constituents, as noted above; thus, the strict adjacency requirement is general to all foci in Romeyka, unlike Pontic Greek.(55) Pontic Greek: aika emorfa peðʝa esis kamian iðeten?
       such beautiful.ACC children.ACC you.NOM ever see.PST.2PL
       ‘Have you ever seen such beautiful children?’

      (Sitaridou and Kaltsa, 2014: p. 15 apud Drettas, 1997: p. 183)

The second difference between Romeyka and Pontic Greek is that the latter has another means of encoding contrastive focus which is not present in Romeyka: the use of discourse particles. This is exemplified by the particles *cela* and *ki*. Both assign contrastive focus to the constituent to which they attach, though differ somewhat in distribution. *Cela* is always in postposition, though never enclitic to the verb; in (56), it appears post-sententially, so contrastively focusing the whole VP. Conversely, *ki* is always enclitic to the verb, for example, contrasting the verbal constituent *eperane = ki* ‘they took’ with the predicate in the second main clause, *eksenkan = aten aso plan tin portant* ‘they forced her through the side door’ in (57) (Sitaridou and Kaltsa, 2014: p. 11).(56) Pontic Greek:
   a. kit eceka ce c^h^ eleps ato cela.
      lie.3SG there and NEG see.2SG it.ACC PRT
      ‘It is there and you don’t even see it.’
   b. efaen do fain atun c edoken atsen cela.
      eat.PST.3SG the.ACC food.ACC their and strike.PST.3SG them PRT
      ‘He ate their food and beat them as well.’

      (Sitaridou and Kaltsa, 2014: p. 11 apud Drettas, 1997: p. 410)(57) Pontic Greek: atos … eperane ci ti marian eksenkan aten
      he take.PST.3PL PRT the.ACC Maria.ACC take.out.PST.3PL her.ACC
      aso plan din bortan.
      from.the.ACC sides.ACC the.ACC door.ACC
      ‘He … they took Maria and forced her to exit through the side door.’

      (Sitaridou and Kaltsa, 2014: p. 11 apud Drettas, 1997: p. 481)

Let us summarise our conclusions from this section. In Romeyka, topics and foci can both be expressed by occurring *ex situ*. In the case of foci, this is obligatory: focussed constituents in Romeyka always occupy the immediate preverbal position, instead of the pragmatically unmarked postverbal position (see §3), no matter the type of focus or syntactic category of the constituent. In the case of topics, there is also an option to occur in a postverbal position, but only for non-contrastive given information; contrastive topics must be preverbal, like foci. Contrastive topics can also occur with a *pa(l)*-particle.

This differs from SMG, which allows non-contrastive information—for example, aboutness topics and information foci—to occur postverbally, and does not exhibit the strict adjacency requirement on preverbal topics/foci. Romeyka also lacks topic particles like *pa(l)*. It is strikingly more like Pontic Greek: like Romeyka, it allows foci and topics of all types to be realised preverbally; and it can also mark contrastive topics by a topic particle, *pa*. There are still differences, however: left dislocation is not obligatory for contrastive foci in Pontic Greek, for example, because focus can instead be marked by a particle; and the *pa*-particle is unlike Romeyka’s *pa(l)* in encoding contrast.

### 4.4 *Wh*-questions and focus

MG displays *wh*-questions, (see (58)). Similarly, Romeyka also employs *wh*-questions (see (59)) (see Michelioudakis and Sitaridou, 2013, 2016):(58) Modern Greek:
   a. pços fílise ti maría? who.NOM kiss.PST.3SG the.ACC Maria.ACC
      ‘Who kissed Maria?’

      (Alexopoulou and Baltazani, 2012)b. pçon fílise i maría?
      whoACC kiss.PST.3SG the.NOM Maria.NOM
      ‘Who did Maria kiss?’

      (Alexopoulou and Baltazani, 2012)(59) Romeyka:
   a. Pĺos eðótšen tin kosːáran?who.NOM give.PST.3SG the.ACC hen.ACC
      ‘Who gave the hen?
      (S01; 812_0093; 00:03)
   b. χavítsæ Pĺos éfaen? pudding.ACC who.NOM eat.PST.3SG
      ‘Who ate puddings?
      (S01; 812_0057; 04:06)
   c. alís DÓɣna ðótšen?
      Alis.NOM  what.ACC give.PST.3SG
      ‘What did Alis give?’
       (S01; 812_0093; 00:16)

Crucially, the order of *wh*-questions is strictly order-preserving in Romeyka (see (60) and (61)) (see Michelioudakis and Sitaridou, 2013, 2016):(60) Romeyka:
   a. Pĺos eðótšen tin kosːáran? who.NOM give.PST.3SG the.ACC hen.ACC
      ‘Who gave the hen?
      (S01; 812_0093; 00:03)
   b. ?Pĺos tin kosːáran eðótšen? who.NOM the.ACC hen.ACC give.PST.3SG
       ‘Who gave the hen?
(61) Romeyka:
   a. χavítsæ Pĺos éfaen? puddings.ACC who.NOM eat.PST.3SG
      ‘Who ate puddings?’
      (S01; 812_0057; 04:06)
   b. ?Pĺos χavítsæ éfaen?
      who.NOM puddings.ACC eat.PST.3SG
      ‘Who ate puddings?’

*wh*-phrases are obligatorily left-dislocated (see (62)), with no option but to leave any *wh*-phrase *in situ* (Michelioudakis and Sitaridou, 2013, 2015):(62) Romeyka:
   a. alís DÓɣna ðótšen?
      Alis.NOM  what.ACC give.PST.3SG
      ‘What did Alis give?’
      (S01; 812_0093; 00:16)
   b. ?alís ðótšen DÓɣna?
      Alis.NOM  give.PST.3SG what.ACC
       ‘What did Alis give?’

In this section, we have shown that *wh*-phrases in *wh*-questions in Romeyka occupy the same position that focussed constituents occupy.

## 5 Information structure: clausal architecture of the left periphery

Having established the distributions for topics and foci, we currently move to consider what clausal structure is required to model Romeyka’s topics and foci, adopting a cartographic perspective on information structure (cf. Neocleous, 2020: ch. 5 for a minimalist alternative). First, in §5.1, we consider the *number* of topic positions required in view of the data discussed in §4. We then map these positions onto a functional hierarchy for the clausal left periphery in §5.2, contrasting our proposal with the information structure systems of Pontic Greek and Turkish, respectively.

### 5.1 How many topic positions are there in Romeyka?

Given the evidence above, it is clear there are two positions in which given information, i.e., topics, may occur in Romeyka: a preverbal position; and a postverbal position. Note that this raises the following question: if *both* the preverbal and the postverbal domain can accommodate given information, then what differentiates these interpretatively? As we have already shown, contrastive given information can only ever appear in the preverbal domain—it is infelicitous in the postverbal domain. Thus, what differentiates the preverbal from the postverbal topics is the [contrast] feature; the preverbal, but not postverbal, encodes [contrast] to some extent at least.

The examples in (63) and (64) provide additional evidence to this effect: in (63), the object *tin aišén* ‘Ayşe’ carries [non-contrastive] given information and can occur felicitously in both preverbal (63)a and postverbal (63)b position, whereas the object *dolmán* ‘dolma’ in (64) can only appear preverbally (64)a, but not postverbally (64)b, by virtue of carrying [contrastive] given information.(63) Romeyka:
   a. [tin aišén]_A-Top_ o dohtóris epíren.
      the.ACC Ayşe.ACC the.NOM doctor.NOM marry.PST.3SG
      ‘The doctor married Ayşe.’
   b. o dohTÓris epíren [tin aišén]_A-Top_. the.NOM doctor.NOM marry.PST.3SG the.ACC Ayşe.ACC
      ‘The doctor married Ayşe.’
      (S01; 140102_0008; 01:15)
(64) Romeyka:
   a. [dolmán]_C-Top_ o mehmétis éfaen. dolma.ACC the.NOM Mehmetis.NOM eat.PST.3SG
      ‘Mehmetis ate dolma.’
      (S01; 150702_0014; 11:46)
   b. #o mehMÉtis éfaen [dolmán]_C-Top_. the.NOM Mehmetis.NOM eat.PST.3SG dolma.ACC
      ‘Mehmetis ate dolma.’

It is important to note that we can have multiple TopP in Romeyka (see (65)):(65) Romeyka:
      [eɣó]_A-Top_ [ton p^h^ará pal]_C-Top_ ðíɣo se.
      I the.ACC money.ACC PRT give.1SG you.ACC
      ‘I give you the money.’
      (S01; 0120713192027; 01:49)

### 5.2 Clausal architecture of the left periphery

We are now able to propose an architecture for the left periphery of the Romeyka clause. Adopting a cartographic perspective, we take the focus and topic positions identified above to be realised by projections in the functional structure of the left periphery. It is worth noting at this stage that we take the external argument to raise to a high left-peripheral position in Romeyka, namely, the specifier of a (potentially iterated) TopP (for arguments to this effect, see Neocleous, 2020: pp. 105–110); this explains the ability of a topicalised/focussed object to target the left periphery but still follow the subject.

To determine how the relevant topic/focus positions, i.e., projections, are arranged hierarchically, we apply the tests used by Neeleman and van de Koot (2008) and Şener (2010) in their investigations of the information structure of Dutch and Turkish, respectively.

Consider first example (66). The context in (66)a favours an interpretation of the subject in (66)b-c, *o mehmétis* ‘Mehmetis’, as a contrastive topic, as it is the constituent which forms the expected answer. On the other hand, the object in (66)b-c, *dolmán* ‘dolma’, is interpreted as a contrastive focus. This follows from the well-known observation that, in answers to *wh*-questions, the constituent corresponding to the *wh*-operator is typically focussed (e.g., Neocleous, 2020: p. 114, Michelioudakis and Sitaridou, 2013, 2016).(66) Romeyka:
   a. Question:
      alís do epítšen?
      Alis.NOM what.ACC do.PST.3SG
      do éfaen so bairámin?
      what.ACC eat.PST.3SG in.the.ACC Bayram.ACC
      ‘What did Alis do? What did he eat at Bayram?’
      Answers:
   b. válːahi, utš ekséro alís do epítšen, áma … frankly NEG know.1SG Alis.NOM what.ACC do.PST.3SG but …
      ‘Frankly, I don’t know about Alis, but …’
   c. [o mehmétis]_C-Top_ [dolmán]_C-Foc_ éfaen. the.NOM Mehmetis.NOM dolma.ACC eat.PST.3SG
      ‘Mehmetis ate dolma.’
      (S01; 150702_0014; 09:06)
      d. #[dolmán]_C-Foc_ [o mehmétis]_C-Top_ éfaen. dolma.ACC the.NOM Mehmetis.NOM eat.PST.3SG
      ‘Mehmetis ate dolma.’

Importantly, there is a contrast between the felicitous (66)b in which the contrastive focussed constituent (C-Foc) *follows* the contrastive topic (C-Top), and the infelicitous (66)c in which C-Foc *precedes* C-Top. In other words, C-Top > C-Foc order is felicitous; C-Foc > C-Top is not.

This restriction holds even when we reverse the relation between grammatical function and information structure. The context in (67) is set up to favour an interpretation of the *object* as C-Top and the *subject* as C-Foc, the opposite of (66).(67) Romeyka:
   a. Question:
      o tšorbás do eʝéndo?
      the.NOM soup.NOM what.ACC happen.PST.3SG
      atón kanís éfaen æ?
      this.ACC anyone.NOM eat.PST.3SG it.ACC
      ‘What about the soup? Has anyone eaten it?’
      Answers:
   b. válːahi, utš eksér o tšorbás do eʝéndo, frankly NEG know.1SG the.NOM soup.NOM what.ACC happen.PST.3SG
      áma …
      but …
      ‘Frankly, I don’t know about the soup, but …’
   c. [dolmán]_C-Top_ [o mehMÉtis]_C-Foc_ éfaen. dolma.ACC the.NOM Mehmetis.NOM eat.PST.3SG
      ‘Mehmetis ate dolma.’
      (S01; 150702_0014; 11:46)
      d. #[o mehMÉtis]_C-Foc_ [dolmán]_C-Top_ éfaen. the.NOM Mehmetis.NOM dolma.ACC eat.PST.3SG
      ‘Mehmetis ate dolma.’

Nonetheless, C-Top > C-Foc is again the only felicitous order as in (67)b, with C-Foc > C-Top in (67)c being infelicitous.

Third and finally, this same restriction holds for the interaction between VP-internal objects, too. The sentences in example (68) contain a ditransitive verb, where the context is set up to favour the interpretation of the IO as C-Foc and the DO as C-Top. Moreover, again, only the C-Top > C-Foc order (that is, DO > IO) order in (68)b is felicitous; C-Foc > C-Top (IO > DO) is not (68)c.(68) Romeyka:
   a. Question:
      i antíka tše i sandália do
      the.NOM antique.NOM and the.NOM chair.NOM what.ACC
      eʝéndo? o pápʰos tínan éðocen æ?
      happen.PST.3SG the.NOM grandfather.NOM who.ACC give.PST.3SG it.ACC
      ‘What about the antique table and the chair? Who did your granddad bequeath them to?’
      Answers:
   b. válːahi i antíka do eʝéndo frankly the.NOM antique.NOM what.ACC happen.PST.3SG
      utš ekséro, áma …
      NEG know.1SG but …
      ‘Frankly, I don’t know about the antique table, but …’
   c. [ti sandalían]_C-Top_ [ton TŠÍri m]_C-Foc_ eðótšen. the chair.ACC the father.ACC I.POSS give.PST.3SG
       ‘my granddad bequeathed the chair to my dad.’
       (S01; 150702_0014; 14:01)
      d. #[ton TŠÍri m]_I-Foc_ [ti sandalían]_C-Top_ eðótšen. the father.ACC I.POSS the chair.ACC give.PST.3SG
      ‘my granddad bequeathed the chair to my dad.’

Moreover, in (69), where we reverse the mapping of grammatical function to information structure, such that the IO is currently interpreted as C-Top, and the DP as C-Foc, the ordering restriction still holds. This is true even though it reverses the felicity contrast as it relates to DO and IO compared to (68): currently, IO > DO is the only felicitous order, with DO > IO illicit—the opposite of (68).(69) Romeyka:
   a. Question:
      o tšíris DO eʝéndo?
      the.NOM father.NOM what.ACC happen.PST.3SG
      o pápʰos DO  éðocen aton?
      the.NOM grandfather.NOM what.ACC give.PST.3SG he.ACC
      ‘What about your dad? What has granddad bequeathed to him?’
      Answers:
   a. válːahi o tšíris m DO eʝéndo
      frankly the.NOM father.NOM I.POSS what.ACC happen.PST.3SG
      utš ekséro, áma …
      NEG know.1SG but
      ‘Frankly, I don’t know about my dad, but …’
   b. [ti mána m]_C-Top_ [to saÁtʰin]_C-Foc_ efítšen. the mother.ACC I.POSS the watch.ACC bequeath.PST.3SG
      ‘my granddad bequeathed the watch to my mother.’
      (S01; 150702_0023; 08:18)
   c. #[to saÁtʰin]_C-Foc_ [ti mána m]_C-Top_ efítšen. the watch.ACC the mother.ACC I.POSS bequeath.PST.3SG
      ‘my granddad bequeathed the watch to my mother.’

Thus, there is robust evidence for the generalisation that C-Top precedes C-Foc in Romeyka, independent of the grammatical functions the relevant constituents bear.

Indeed, the generalisation can be broadened to range over *all* foci. We have already established that information focus occupies the same position as contrastive focus. Accordingly, I-Foc follows contrastive topics just like C-Foc does (see (70)); and follows aboutness topics (see (71)). This strongly suggests a clausal architecture for Romeyka whereby the single dedicated Focus projection follows the (C-)Topic projection, Top > Foc.(70) Romeyka:
   a. Question: tsi birʝýlis t aðélfæ
      the.GEN Birgül.GEN the.NOM brothers.NOM
      d epíkane so pártin?
      what.ACC do.PST.3PL at.the.ACC party.ACC
      ‘What did Birgül’s brothers get to drink at the party?’
      Answers:
   a. válːahi as aðélfæ tes utš ekséro, áma …
      frankly from.the.ACC brothers.ACC she.POSS NEG know.1SG but …
      ‘Frankly, I do not know about all her brothers, but …’
   b. [úlːunon o mikrón]_C-Top_ [raCÍN]_I-Foc_ epíen. all.GEN the.NOM young.NOM raki.ACC drink.PST.3SG
      ‘Birgül’s youngest brother drank raki.’
      (S01; 150702_0023; 23:26)
   c. #[raCÍN]_I-Foc_ [úlːunon o mikrón]_C-Top_ epíen. raki all.GEN the.NOM young.NOM drink.PST.3SG
      ‘Birgül’s youngest brother drank raki.’
(71) Romeyka:
   a. Question:
      avúto to faín PÍon patsín epítšen?
      this.ACC the.ACC food.ACC which.NOM girl.NOM make.PST.3SG
      ‘Which girl made this food?’
      (S01; 150703_0042; 03:32)
   b. Answer:
      [avúton to faín]_A-Top_ [i miNÉ] _I-Foc_ epítšen.
      this.ACC the.ACC food.ACC the.NOM Mine.NOM make.PST.3SG
      [t álːon]_C-Top_ [i aiŠÉ]_C-Foc_ epítšen.
      the.ACC other.ACC the.NOM Ayşe.NOM make.PST.3SG
      ‘Mine made this food; Ayşe made the other one.’
      (S01; 150703_0042; 03:45)

Interestingly, this pattern also holds in Turkish. Like Romeyka, the focussed constituent in Turkish is argued to be placed immediately preverbally, no matter what sub-type of focus it conveys (see Göksel and Kerslake, 2005; Kornfilt, 1997; Şener, 2010, i.a.). This is obligatory: nothing that bears information or contrastive focus can be placed in the postverbal field; it must occur immediately preverbally (see Erguvanlı, 1984; Göksel and Kerslake, 2005; Kornfilt, 1997; Şener, 2010, i.a.).^3^ As a consequence, just like Romeyka, contrastive focus cannot precede a contrastive topic in Turkish (since it no longer immediately precedes the verb) (see (72)):(72) Turkish:
   a. Question:
      Can’dan n’aber? O ne yedi partide?
      ‘What about John? What did he eat at the party?’
      Answers:
   b. Valla Can-‘ı bil-mi-yor-um, ama … frankly Can-ACC know-NEG-PROG-1SG but
      ‘Frankly, I don’t know about John, but …’
   c. [Aylın]_C-Top_ [dolma-lar-dan]_C-Foc_ ye-di.
      Aylin-NOM dolma-PL-ABL eat-PST-3SG
      ‘Aylin ate from the dolmas.’
      d. #[dolma-lar-dan]_C-Foc_ [Aylın]_C-Top_ ye-di. dolma-PL-ABL Aylin-NOMeat-PST-3SG
      ‘Aylin ate from the dolmas.’

      (Şener, 2010: p. 19)

In the same way, as information focus must also be immediately preverbal, it must follow contrastive topics in Turkish too—again, parallel to Romeyka (see (73)):(73) Turkish:
   a. Question:
      Filiz-in kardeş-ler-i ne iç-ti parti–de?
      Filiz-GEN sister-PL-POSS what drink-PST-3SG party-LOC
      ‘What did Filiz’s sisters get to drink at the party?’
      Answers:
      Valla tüm kardeş-ler-den haberim yok, ama …
      frankly all sister-PL-ABL news-POSS-1SG NEG but
      ‘Frankly, I do not know about all the sisters but …’
   b. [Filiz-in en küçük kardeş-i]_C-Top_ [rakı-dan]_I-Foc_ iç-ti.
      Filiz-GEN most young sister-3SG-POSS rakı-ABL drink-PST-3SG
      ‘Filiz’s youngest sister drank (from the) rakı.’
   c. #[rakı-dan]_I-Foc_ [Filiz-in en küçük kardeş-i]_C-Top_ iç-ti.
      rakı-ABL Filiz-GEN most young sister-3SG-POSS drink-PST-3SG
      ‘Filiz’s youngest sister drank (from the) rakı.’

      (Şener, 2010: p. 35)

To summarise, Romeyka obeys the hierarchy in (74): topics always precede foci.(74) Hierarchy of discourse features in Romeyka:
   a. Topic > Focus
   b. #Focus > Topic

Given our conclusion that there is a single focus position, and a single preverbal topic position (alongside a postverbal position for non-contrastive given information), this suggests the hierarchy of discourse features in (75):(75) Articulation of discourse-related features in Romeyka:
      A-/C-TopicP I-/C-FocusP TP Given (non-contrastive) information

Given this hierarchy, [topic] and [focus] constitute autonomous structural notions in Romeyka: there is a dedicated left-peripheral projection encoding [topic], and another encoding [focus].

This represents a break from SMG, where the *ex situ*, preverbal position is generally associated with contrastive constituents (either topics and foci), but not information focus which favours a postverbal realisation. Instead, it appears that Romeyka has partially converged on the Turkish pattern: it exhibits the same immediately preverbal focus position regardless of focus type; and has the same restriction that foci must follow topics. However, when it comes to contrastive subject foci Romeyka deviates from Turkish either by having the contrastive focussed subject immediately to the left of the verb and the object higher or postverbally.

This pattern is highly suggestive, in that as it may reflect Romeyka’s sociohistorical profile. Romeyka, as noted above, is the last Asia Minor Greek variety still spoken in Turkey; the speech community, by virtue of being Muslim, was exempted from the forced population exchange of 1923 which followed the cessation of the Greek-Turkish War (1919–1922). Consequently, it has undergone centuries-long contact with Turkish and concomitantly isolation from Standard Modern Greek. The parallels between the Turkish and Romeyka information structure systems may thus instantiate a contact effect.

The comparison with Pontic Greek is also informative in this regard. Pontic Greek is spoken primarily in Greece, and it too has been in some contact with Turkish prior to 1923 and significant contact (and thus attrition) with SMG since then. Interestingly, both Pontic varieties have converged in certain regards on the Turkish pattern, as noted: both have *ex situ* realisations for foci regardless of semantic type, for example. This may reflect parallel outcomes of Turkish contact. However, the organisation of their information structure also differs in significant ways: [contrast], for example, is an autonomous structural notion, with its own dedicated ContrastP projection realised by the *pa*-particle in Pontic Greek, whereas it seems to be a subfeature of [topic]/[focus] instead in Romeyka; and Pontic Greek does not observe the restriction that contrastive foci appear immediately preverbally. This may diagnose a difference in the contact profiles of Romeyka and Pontic Greek since their split (the latter to be taken the Islamisation onset, see Sitaridou, 2014a). The topic clearly awaits further investigation.

## 6 Conclusion

In this paper, we have sought to expand the coverage of formal work regarding the Asia Minor Greek variety, Romeyka, by investigating its word order and information structure. Regarding the latter, we have presented evidence that Romeyka patterns with its cognate variety, Pontic Greek, and Standard Modern Greek in having pragmatically unmarked, underlying VO word order, as well as verb-raising. However, Romeyka also exhibits frequent OV orders, attributable to information structural effects. As a result, we have argued that the organisation of Romeyka’s information structure differs radically from SMG: topics and foci, of all semantic types, are realised *ex situ*, with no association with contrast (unlike SMG). We thus conclude that [topic] and [focus] are autonomous structural notions in Romeyka, realising heads in the clausal left periphery. Romeyka patterns instead with Turkish, which also has *ex situ* topics and foci, and like Romeyka limits them to the immediate preverbal position. Interestingly, the information structure in Romeyka’s closest cognate variety, Pontic Greek, diverges in a significant way from both Romeyka and SMG in having a dedicated ContrastP projection (see Sitaridou and Kaltsa, 2014) absent in Romeyka. The patterns in Romeyka and Pontic Greek may reflect subtly different patterns of contact with Turkish, though a more detailed investigation remains a goal for future inquiry (but see Sitaridou, 2022).

## Data Availability

The datasets presented in this article are not readily available because anonymisation of the data is not available yet. Requests to access the datasets should be directed to is269@cam.ac.uk.

## Footnotes

^1^Throughout the article, we use the term “Romeyka” to refer to the Greek variety, which is spoken in Turkey’s Black Sea today, whereas we use the term “Pontic Greek” to refer to the Pontic Greek variety which is spoken in Greece today. For glossonymy, see Sitaridou (2023a, 2023b) and references therein.

^2^See also Neocleous (2020) which is using the same corpora as the ones detailed here. It is duly noted that his PhD Supervisor, Prof Ioanna Sitaridou, has generously shared her corpus with him, for the exclusive purpose of writing up his thesis and the co-publication of any papers deriving from the thesis.

^3^An anonymous reviewer noted that the immediate preverbal position is not the only position focus can appear in Turkish. It is possible to find non-preverbal focused constituents in Turkish with discourse-given elements intervening in between the focus and the verb (cf. Göksel and Özsoy, 2000; Gürer, 2020, among others).For example, Ali in (iB) and I in (iiB) below bear focus: (i)  A: Yemek-ler-i kim pişir-di? (Ayşe mi Ali mi?)  dish-PL-ACC who cook-PST (Ayşe Q Ali Q)  ‘Who cooked the dishes? (Ali or Ayşe?)’  B: ALİ yemek-ler-i pişir-di, Ayşe değil.  Ali dish-PL-ACC cook-PST Ayşe not  ‘Ali cooked the dishes, not Ayşe.’ (ii) A: Kim ben-i çok sev-iyor?  who I-ACC much love-IPFV-1SG  ‘Who loves me so much?’  B: BEN sen-i çok sev-iyor-um.  I you-ACC much love-IPFV-1SG  ‘I love you so much.’ Indeed, this is possible, but, crucially, in this case the focalised constituent is the subject which appears higher than the object which occupies the position immediately to the left. So, when contrastive foci are subjects they cannot be adjacent to the verb because of the OV nature of the language whereby objects would have to occupy the immediate preverbal position (see Michelioudakis and Sitaridou (2020: p. 247) on the difficulty of diagnosing Multiple Wh-fronting in an OV language). Although we have seen in (67d) that this is not possible in Romeyka (and therefore, Romeyka and Turkish differ in this respect), similar effect is obtained in Romeyka when both the subject and object are new information foci (see Michelioudakis and Sitaridou, 2016: p. 9): (iii) A: pios tinan endže?  who whom bring.PST.3SG  ‘Who brought (what) to whom?’ (iv) B: o yusufis tin aiše ɣalemin endže.  the.NOM Yusufis the.acc Ayşe pencil bring.PST.3SG  ‘Yusuf brought a pencil to Ayşe.’

## Glossary

ABL: ablative
ACC: accusative
FUT: future
GEN: genitive
IMP: imperative
IPFV: imperfective
LOC: locative
MOD: modal
NEG: negation
NOM: nominative
PRT: particle
PL: plural
POSS: possessive
PST: past
SG: singular
A-TOP: aboutness topic
C-Topic: contrastive topic
C-FOC: contrastive focus
DP: determiner phrase
FOC: focus
I-FOC: information focus
NP: noun phrase
PG: Pontic Greek
O: object
SMG: Standard Modern Greek
S: subject
TOP: topic
TP: tense phrase
V: verb
VP: verb phrase
vP: light verb phrase

## References

[ref1] Agouraki Y. (2010). It-clefts and stressed operators in the preverbal field of Cypriot Greek. Lingua 120, 527–554. doi: 10.1016/j.lingua.2008.10.011

[ref3] Alexiadou A. Anagnostopoulou E. (1998). Parametrizing AGR: word order, V movement and EPP-checking. Nat. Lang. Linguist. Theory 16, 491–539. doi: 10.1023/A:1006090432389

[ref9001] Alexiadou A. Anagnostopoulou E. (2000). “Clitic Doubling and (Non-) Configurationality.” Proceedings of NELS 30, 17–28.

[ref9002] Alexopoulou T. Baltazani M. (2012). Focus in Greek wh-questions. In: Kučerová I, Neeleman A, eds. Contrasts and Positions in Information Structure. Cambridge University Press, 206–246.

[ref5] Beaver D. Clark B. (2008). Sense and sensitivity. Oxford: Blackwell.

[ref6] Biberauer T. Roberts I. (2010). “Subjects, Tense and verb-movement” in Parametric variation: Null subjects in minimalist theory. eds. Biberauer T. Holmberg A. Sheehan M. Roberts I. (Cambridge: Cambridge University Press), 263–302.

[ref7] Büring D. (2003). On D-trees, beans, and B-accents. Linguist. Philos. 26, 511–545. doi: 10.1023/A:1025887707652, PMID: 38124636

[ref8] Büring D. (2009). “Towards a typology of focus realization” in Information structure. eds. Zimmermann M. Fery C. (Oxford: Oxford University Press), 177–205.

[ref9] Chatzikyriakidis S. Michelioudakis D. Spathas G. (2015). Greek focus operators and their associates. Studies for the Greek language, vol. 35. Thessaloniki: Aristotle University of Thessaloniki, 167–179.

[ref10] Chomsky N. (1972). Studies on semantics in generative grammar. The Hague: Mouton.

[ref11] Chomsky N. (1982). Some concepts and consequences of the theory of government and binding. Cambridge, MA: MIT Press.

[ref12] Chomsky N. (2001). “Derivation by phase” in Ken Hale: a life in language. ed. Kenstowicz M. (Cambridge, MA: MIT Press), 1–53.

[ref13] Dawkins R. M. (1916). Modern Greek in Asia minor: A study of the dialects of Sílli, Cappadocia and Phárasa with grammar, texts, translations and glossary. Cambridge: Cambridge University Press.

[ref14] Deffner M. (1878). Die Infinitive in den pontischen Dialekten und die zusammengesetzten Zeiten im Neugriechischen. Berlin: Monatsberichte der Königlich Preussischen Akademie der Wissenschaften zu Berlin.

[ref15] Drettas G. (1997). Aspects pontiques. Paris: Association de recherches pluridisciplinaires.

[ref16] Erguvanlı E. (1984). The function of word order in Turkish grammar. Berkeley: University of California Press.

[ref17] Frascarelli M. Hinterhölzl R. (2007). “Types of topics in German and Italian” in On information structure, meaning and form. eds. Schwabe K. Winkler S. (Amsterdam and Philadelphia: Benjamins), 87–116.

[ref18] Göksel A. Kerslake C. (2005). Turkish: A comprehensive grammar. London: Routledge.

[ref19] Göksel A. Özsoy A. S. (2000). “Is there a focus position in Turkish?” in Studies on Turkish and Turkic languages. eds. Göksel A. Kerslake C. (Harrasowitz: Wiesbaden), 219–228.

[ref20] Gryllia S. (2008). On the nature of preverbal focus in Greek: a theoretical and experimental approach (Vol. LOT dissertation series 200). Landelijke: Netherlands Graduate School of Linguistics.

[ref21] Gundel J. K. (1998). On three kinds of focus. In Bosch P. Sandt R. van der, Focus: linguistic, cognitive and computational perspectives (pp. 293–305). Cambridge: Cambridge University Press.

[ref22] Gürer A. (2020). Information structure within interfaces: Consequences for the phrase structure. Berlin: De Gruyter Mouton.

[ref23] Holmberg A. (2005). Is there a little pro? Evidence from Finnish. Linguist. Inq. 36, 533–564. doi: 10.1162/002438905774464322

[ref24] Jackendoff R. (1972). Semantic interpretation in generative grammar. Cambridge, MA: MIT Press.

[ref25] Kisilier M. (2007). Word-order patterns in the Greek dialect of Mariupolis. Proceedings of the 3rd International Conference on Modern Greek Dialects and Linguistic Theory, Nicosia.

[ref26] Koeneman O. Zeijlstra H. (2014). The rich agreement hypothesis rehabilitated. Linguist. Inq. 45, 571–615. doi: 10.1162/LING_a_00167

[ref27] Kornfilt J. (1997). On rightward movement in Turkish. In Johanson L. (Ed.), He Mainz Meeting, Proceedings of the Seventh International Conference on Turkish Linguistics (pp. 107–123). Wiesbaden: Harrassowitz.

[ref28] Krifka M. (2007). “The semantics of questions and the focusation of answers” in Topic and focus, crosslinguistic perspectives on meaning and intonation. eds. Lee C. Gordon M. Büring D. (Dordrecht: Springer), 139–150.

[ref30] Lambrecht K. (1994). Information structure and sentence form. Cambridge: Cambridge University Press.

[ref31] Mackridge P. (1987). Greek-speaking Moslems of north-East Turkey: prolegomena to a study of the ophitic sub-dialect of Pontic. Byz. Mod. Greek Stud. 11, 115–137. doi: 10.1179/030701387790203037

[ref32] Mackridge P. (1999). “The Greek spoken in the region of Pontus” in Dialect enclaves of the Greek language. eds. Christidis A. F. Arapopoulou M. Giannoulopoulou G. (Athens: Hellenic Republic, Ministry of National Education and Religious Affairs), 101–105.

[ref33] Melanofrydis P. (2001). Οι Κλωστοί. Θεσσαλονίκη: Αδελφοί Κυριακίδη.

[ref34] Michelioudakis D. Sitaridou I. (2012). “Syntactic microvariation: dative constructions in Greek” in Datives in variation: A micro-comparative perspective. eds. Etxepare R. Fernández B. (Oxford: Oxford University Press), 212–255.

[ref35] Michelioudakis D. Sitaridou I. (2013). Multiple wh-fronting in Romeyka. In Ralli A. Joseph B. Janse M. (Ed.), Proceedings of the Fifth International Conference of Modern Greek Dialects and Linguistic Theory (MGDLT5), Ghent, 20–22 September 2012, (pp. 353–378).

[ref36] Michelioudakis D. Sitaridou I. (2016). Recasting the typology of multiple wh-fronting: evidence from Pontic Greek. Glossa 1, 40–72. doi: 10.5334/gjgl.72

[ref37] Michelioudakis D. Sitaridou I. (2020). “Towards a formal model of transfer under contact: contrasting Asia minor Greek to mainland Greek and Turkish in search of syntactic borrowings” in Theoretical approaches to contrastive linguistics: Morphological and syntactic perspectives. eds. Georgiafentis M. Giannoulopoulou G. Koliopoulou M. Tsokoglou A. (London: Bloomsbury Studies in Theoretical Linguistics), 246–261.

[ref38] Molnár V. (2006). “On different kinds of contrast” in Architecture of focus. Studies in generative grammar. eds. Molnár V. Winkler S., vol. 82 (Berlin/New York: Mouton de Gruyter), 197–233.

[ref39] Neeleman A. van de Koot H. (2008). Dutch scrambling and the nature of discourse templates. J. Comp. Ger. Linguist. 11, 137–189. doi: 10.1007/s10828-008-9018-0

[ref40] Neocleous N. (2020). Word order and information structure in Romeyka: A syntax and semantics interface account of order in a minimalist system: PhD dissertation, University of Cambridge.

[ref41] Neocleous N. (2022). “The evolution of VO and OV alternation in Romeyka” in *Word order variation – Semitic, Turkic, Caucasian and indo-European languages in contact*. STUF – language typology and universals. In Jügel, Thomas, and Hiwa Asadpour, eds., Word Order Variation : Semitic, Turkic and Indo-European Languages in Contact, De Gruyter, Berlin, Germany.

[ref42] Neocleous N. Sitaridou I. (2022). Never just contact. The rise of final auxiliaries in Asia minor Greek. Diachronica 39, 369–408. doi: 10.1075/dia.17048.neo, PMID: 33486653

[ref43] Özkan H. (2013). The Pontic Greek spoken by Muslims in the villages of Beşköy in the province of present-day Trabzon. Byz. Mod. Greek Stud. 37, 130–150. doi: 10.1179/0307013112Z.00000000023

[ref45] Parcharidis I. (1880). Γραμματικὴ τῆς διαλέκτου Τραπεζοῦντος: Περὶ ἐπιθέτων, ἀντωνυμιῶν και βαρυτόνων ρημάτων, vol. Χειρόγραφο 335. Athens: Κέντρο Ἐρεύνης τῶν Νεοελληνικῶν Διαλέκτων και Ἰδιωμάτων, Ἀκαδημία Αθηνών.

[ref46] Pollock J.-Y. (1989). Verb-movement, universal grammar, and the structure of IP. Linguist. Inq. 20, 365–424. Stable URL: http://www.jstor.org/stable/4178634

[ref47] Roberts I. (1993). Verbs and diachronic syntax: A comparative history of English and French. Dordrecht: Kluwer.

[ref48] Rooth M. E. (1992). A theory of focus interpretation. Nat. Lang. Semant. 1, 75–116. doi: 10.1007/BF02342617

[ref49] Sağlam E. (2017). Constitutive ambiguities. Subjectivities and memory in the case of Romeika-speaking communities of Trabzon: Unpublished PhD dissertation, Birkbeck, University of London.

[ref50] Schreiber L. Sitaridou I. (2017). Assessing the sociolinguistic vitality of Istanbulite Romeyka: an attitudinal study. J. Multiling. Multicult. Dev. 39, 1–16. doi: 10.1080/01434632.2017.1301944

[ref51] Şener S. (2010). (Non-)peripheral matters in Turkish syntax: Unpublished PhD dissertation, University of Connecticut.

[ref52] Sitaridou I. (2013). “Greek-speaking enclaves in Pontus today: the documentation and revitalization of Romeyka” in Keeping languages alive. 322 word order and information structure in Romeyka: A syntax and semantics interface account of order in a minimalist system language endangerment: Documentation, pedagogy and revitalization. eds. Jones M. C. Ogilvie S. (Cambridge: Cambridge University Press), 98–112.

[ref53] Sitaridou I. (2014a). The Romeyka infinitive: continuity, contact and change in the Hellenic varieties of Pontus. Diachronica 31, 23–73. doi: 10.1075/dia.31.1.02sit

[ref54] Sitaridou I. (2014b). Modality, antiverdicality and complementation: the Romeyka infinitive as a negative polarity item. Lingua 148, 118–146. doi: 10.1016/j.lingua.2014.05.017

[ref55] Sitaridou I. (2015). Reframing the Phylogeny of Asia Minor Greek: The View from Pontic Greek.” CHS Research Bulletin 4, no.1. http://nrs.harvard.edu/urn-3:hlnc.essay:SitaridouI.Reframing_the_Phylogeny_of_Asia_Minor_Greek.2016.

[ref56] Sitaridou I. (2017a). “The rise of novel negators in Romeyka: negative existential cycle and monoclausality”, Workshop on the negative existential cycle from a historical-comparative perspective, Stockholm University, 04–5/05/2017.

[ref57] Sitaridou I. (2017b). “Null objects in Romeyka: contact with Turkish or internal conditioning?”, Talk at the Workshop on historical language contact in English and beyond, Aristotle University of Thessaloniki, 31/03–02/04/2017.

[ref58] Sitaridou I. (2021). “(in)vulnerable inflected infinitives as complements to volitionals and modals: evidence from Galician and Romeyka” in Syntactic features and the limits of syntactic change. eds. Eythrsson T. Nsson J. G. J. (Oxford: Oxford University Press), 161–177.

[ref59] Sitaridou I. (2022). “On the redundancy of a theory of language contact: cue-based reconstruction in a sociolinguistically informed manner” in Language change: Theory and methodologies in the 21st century. eds. Lavidas N. Nikiforidou K. (Leiden: Brill), 15–52.

[ref60] Sitaridou I. (2023a). “Romeyka” in Endangered Languages in Turkey. eds. Bagriacik M. Demirok O. Ozturk B. (Istanbul: The Laz Instituite), 118–146.

[ref61] Sitaridou I. (2023b). Romeyka. “Türkiye'nin tehlike altındaki dilleri” içinde, Bağrıaçık Metin Demirok Ömer ve Öztürk Balkız (Ed.), İstanbul: Laz Enstitüsü.

[ref9003] Sitaridou I. (2023c). “Key Insights about Romeyka’s Linguistic Description and Evolution, Vitality, and Revitalisation Prospects After 15 Years of Field-Based Research”. Translated byJerneja Kavčič. Keria: Studia Latina Et Graeca 25, 9–32. doi: 10.4312/keria.25.2.9-32

[ref62] Sitaridou I. (2024). Τα ρομέικα χθες και σήμερα. Romeyka exhibition catalogue. Octana: Thessaloniki, 26–54.

[ref63] Sitaridou I. (in prep.). The evolution of negation in Romeyka: Ms, University of Cambridge.

[ref64] Sitaridou I. Kaltsa M. (2014). Contrastivity in Pontic Greek. Lingua 146, 1–27. doi: 10.1016/j.lingua.2014.04.005

[ref65] Tsimpli I. M. (1995). “Focusing in modern Greek” in Discourse configurational languages. ed. Kiss K. E. (Oxford: Oxford University Press), 176–207.

[ref66] van der Wal J. (2016). Diagnosing focus. Stud. Lang. 40, 259–301. doi: 10.1075/sl.40.2.01van, PMID: 33486653

[ref67] Vikner S. (1995). Verb movement and expletive subjects in the Germanic languages. Oxford: Oxford University Press.

[ref68] Vikner S. (1997). “V-to-I movement and inflection for person in all tenses” in The new comparative syntax. ed. Haegeman L. (London: Longman), 189–213.

